# Effect of Repeated Low-level Red Light on Myopia Prevention Among Children in China With Premyopia: A Randomized Clinical Trial

**DOI:** 10.1001/jamanetworkopen.2023.9612

**Published:** 2023-04-26

**Authors:** Xiangui He, Jingjing Wang, Zhuoting Zhu, Kaidi Xiang, Xinzi Zhang, Bo Zhang, Jun Chen, Jinliuxing Yang, Linlin Du, Chunjin Niu, Mei Leng, Jiannan Huang, Kun Liu, Haidong Zou, Mingguang He, Xun Xu

**Affiliations:** 1 Department of Clinical Research, Shanghai Eye Disease Prevention and Treatment Center, Shanghai Eye Hospital, Shanghai Vision Health Center and Shanghai Children Myopia Institute, Shanghai, China; 2 Department of Ophthalmology, Shanghai General Hospital, Shanghai Jiao Tong University School of Medicine, National Clinical Research Center for Eye Diseases, Shanghai Center of Eye Shanghai Key Laboratory of Ocular Fundus Diseases, Engineering Center for Visual Science and Photomedicine, Shanghai, China; 3 State Key Laboratory of Ophthalmology, Zhongshan Ophthalmic Center, Sun Yat-sen University, Guangzhou, China; 4 Centre for Eye Research Australia, Royal Victorian Eye and Ear Hospital, Melbourne, Victoria, Australia; 5 Division of Ophthalmology, Department of Surgery, University of Melbourne, Melbourne, Victoria, Australia; 6 Department of Ophthalmology Prevention, Changning Center for Disease Control and Prevention, Shanghai, China; 7 Department of Teaching and Research, Changning Institute of Education, Shanghai, China

## Abstract

**Importance:**

Myopia is a global concern, but effective prevention measures remain limited. Premyopia is a refractive state in which children are at higher risk of myopia, meriting preventive interventions.

**Objective:**

To assess the efficacy and safety of a repeated low-level red-light (RLRL) intervention in preventing incident myopia among children with premyopia.

**Design, Setting, and Participants:**

This was a 12-month, parallel-group, school-based randomized clinical trial conducted in 10 primary schools in Shanghai, China. A total of 139 children with premyopia (defined as cycloplegic spherical equivalence refraction [SER] of −0.50 to 0.50 diopter [D] in the more myopic eye and having at least 1 parent with SER ≤−3.00 D) in grades 1 to 4 were enrolled between April 1, 2021, and June 30, 2021; the trial was completed August 31, 2022.

**Interventions:**

Children were randomly assigned to 2 groups after grade stratification. Children in the intervention group received RLRL therapy twice per day, 5 days per week, with each session lasting 3 minutes. The intervention was conducted at school during semesters and at home during winter and summer vacations. Children in the control group continued usual activities.

**Main Outcomes and Measures:**

The primary outcome was the 12-month incidence rate of myopia (defined as SER ≤−0.50 D). Secondary outcomes included the changes in SER, axial length, vision function, and optical coherence tomography scan results over 12 months. Data from the more myopic eyes were analyzed. Outcomes were analyzed by means of an intention-to-treat method and per-protocol method. The intention-to-treat analysis included participants in both groups at baseline, while the per-protocol analysis included participants in the control group and those in the intervention group who were able to continue the intervention without interruption by the COVID-19 pandemic.

**Results:**

There were 139 children (mean [SD] age, 8.3 [1.1] years; 71 boys [51.1%]) in the intervention group and 139 children (mean [SD] age, 8.3 [1.1] years; 68 boys [48.9%]) in the control group. The 12-month incidence of myopia was 40.8% (49 of 120) in the intervention group and 61.3% (68 of 111) in the control group, a relative 33.4% reduction in incidence. For children in the intervention group who did not have treatment interruption secondary to the COVID-19 pandemic, the incidence was 28.1% (9 of 32), a relative 54.1% reduction in incidence. The RLRL intervention significantly reduced the myopic shifts in terms of axial length and SER compared with the control group (mean [SD] axial length, 0.30 [0.27] mm vs 0.47 [0.25] mm; difference, 0.17 mm [95% CI, 0.11-0.23 mm]; mean [SD] SER, –0.35 [0.54] D vs –0.76 [0.60] D; difference, –0.41 D [95% CI, –0.56 to –0.26 D]). No visual acuity or structural damage was noted on optical coherence tomography scans in the intervention group.

**Conclusions and Relevance:**

In this randomized clinical trial, RLRL therapy was a novel and effective intervention for myopia prevention, with good user acceptability and up to 54.1% reduction in incident myopia within 12 months among children with premyopia.

**Trial Registration:**

ClinicalTrials.gov Identifier: NCT04825769

## Key Points

**Question:**

Does a repeated low-level red-light (RLRL) intervention prevent incident myopia among children with premyopia?

**Findings:**

In this randomized clinical trial including 278 school-aged children with premyopia, the incidence of myopia was lower among children receiving RLRL therapy than among controls.

**Meaning:**

Exposure to RLRL is a novel and effective intervention for myopia prevention among children with premyopia, with good user acceptability and safety.

## Teaser

This randomized clinical trial assesses the efficacy and safety of a repeated low-level red-light intervention in preventing incident myopia among children in China with premyopia.

## Introduction

Myopia is one of the most common eye diseases and is of worldwide concern.^1,2,3^ In some parts of East and Southeast Asia, myopia prevalence among high school students reaches 80% to 90%.^2,3,4,5^ Myopia poses a burden on individuals and society for refractive error correction.^6^ In addition, early-onset myopia is more likely to progress to high myopia,^7,8,9^ which significantly increases the risk of potential vision-threatening conditions, such as myopic macular degeneration.^10^ Therefore, myopia prevention is of significance.

Currently, several interventions have been proposed to prevent the onset of myopia, such as increased outdoor time,^11,12,13,14,15,16,17,18,19,20,21,22,23^ reducing activities done at a short working distance,^18,19,20,23,24,25,26,27,28^ Chinese eye exercises,^29,30,31,32,33^ and low-dose atropine.^34,35^ The efficacy rate of these interventions at preventing myopia within 1 year ranged from 11.0% to 54.3%.^11,12,13,14,15,16,17,18,19,20,21,22^

Repeated low-level red-light (RLRL) therapy delivered by a device emitting 650-nm visible red light has been proposed as an alternative myopia control intervention.^36,37,38,39,40,41^ Recent research has reported increased blood flow and stabilization of axial elongation among children after the RLRL intervention, which might suggest that RLRL therapy could ameliorate scleral hypoxia and thus prevent the development of myopia.^36^ Despite that, its efficacy at preventing myopia remains unknown. Given that RLRL intervention requires dedicated devices and an investment of time, the technology is most useful for children who are at high risk of developing myopia. In this trial, we intended to enroll children with premyopia, who were normally considered as having a greater risk of developing myopia,^42,43,44,45,46^ to further assess the efficacy and safety of RLRL therapy for myopia prevention among them.

## Methods

This 12-month, 2-group, single-blinded, school-based randomized clinical trial was conducted in Shanghai, China. Children from 10 primary schools were enrolled between April 1, 2021, and June 30, 2021. Examinations were conducted at baseline and at the 3-, 6-, 9-, and 12-month follow-up visits. This trial was completed August 31, 2022. The study was approved by the Shanghai General Hospital Ethics Committee and adhered to the tenets of the Declaration of Helsinki.^47^ Written informed consent was obtained from participants’ guardians. This trial was registered with ClinicalTrials.gov (NCT04825769) and followed the Consolidated Standards of Reporting Trials (CONSORT) reporting guideline (see the trial protocol in Supplement 1).

### Eligibility Criteria

Eligible participants were primary school students in grades 1 to 4 (aged 6-11 years) with premyopia, defined as a cycloplegic spherical equivalent refraction (SER) of the more myopic eye in the range of −0.50 to 0.50 (inclusive) diopters (D) and having at least 1 parent with an SER in either eye of −3.00 D or less. Children were excluded if they had astigmatism of 1.50 D or more, anisometropia of 1.50 D or more, strabismus and other ocular abnormalities, any systemic diseases, or a history of any myopia interventions.

### Randomization and Masking

The randomization was stratified by grade and allocated to individuals at a ratio of 1:1. The randomization list was generated using R software, version 3.6 (R Group for Statistical Computing). To avoid stigmatization for being selected or not, children, parents, and teachers were educated on the purpose of the study and randomization. They were also informed of the right to withdraw at any time. Children and their guardians were aware of the study allocation due to the nature of the intervention. Outcome assessors and statisticians were masked.

### Intervention

Children in the intervention group received the RLRL intervention, while those in the control group did not. The RLRL intervention was provided by a desktop device (Eyerising, Suzhou Xuanjia Optoelectronics Technology; eFigure 1 in Supplement 2), which consists of semiconductor laser diodes and delivers low-level red light with a mean (SD) wavelength of 650 (10) nm. The device is certified as a class IIa device by the China National Medical Products Administration.^36,37^ The RLRL intervention was conducted twice per day, 5 days per week, with each session lasting 3 minutes and with an interval of at least 4 hours between 2 sessions.

### Intervention Compliance Monitoring

During semesters, children in the intervention group completed treatment at school. During summer and winter vacations, guardians were trained by teachers to supervise the intervention at home. The device was linked to the internet with an automated diary function to record the treatment history. In addition, 2 investigators (X.H. and J.W.) were responsible for the intervention compliance management. School teachers or parents or legal guardians were reminded about the intervention to improve treatment compliance every week.

### Data Collection

Clinical examinations were performed at baseline and at 3-, 6-, 9-, and 12-month follow-up visits. Baseline demographic characteristics were collected via questionnaire; the information was obtained from school teachers with permission from the participants and their guardians.

Uncorrected visual acuity (UCVA) and best-corrected visual acuity (BCVA) were assessed at 4 m by trained optometrists using the Early Treatment Diabetic Retinopathy Study (ETDRS) logMAR chart (Guangzhou Xieyi Weishikang). Axial length (AL) was measured before cycloplegia using the IOLMaster (Carl Zeiss Meditec) and averaged until the maximum error did not exceed 0.05 mm. Cycloplegia was induced half-yearly using 2 drops of 1% cyclopentolate (Alcon) 5 minutes apart. One additional drop was administered 5 minutes later if cycloplegia was insufficient. After an additional 30 minutes, full cycloplegia was present if the pupil diameter reached at least 6 mm and the pupillary reaction to light was absent. Refraction data were measured using an autorefractor (KR8800; Topcon) 3 times after cycloplegia and averaged until the desired precision (0.25 D) was achieved. Optical coherence tomography (OCT) scans and fundus images were obtained from swept-source OCT (DRI OCT Triton; Topcon) under radiographic scanning (radial 9 mm). Choroidal thickness was obtained automatically from the built-in segmentation software.

### Outcomes

The primary outcome was the 12-month cumulative incidence of myopia in both groups. Myopia was defined as the cycloplegic SER (sphere plus half of cylinder) of −0.50 D or less in the more myopic eye. Secondary outcomes of the study were the changes in SER, AL, choroidal thickness, UCVA, and BCVA over 1 year. Data from the more myopic eye at baseline were also used for secondary outcomes analysis.

### Adverse Events

At baseline, all children in the intervention group completed 1 session of the RLRL intervention. The children were asked to close their eyes until the bright spot, also known as the *afterimage*, disappeared, then the duration of the afterimage was documented. If the afterimage duration exceeded 6 minutes, the child was clinically considered too sensitive to the intervention and was excluded (n = 2). In addition, eye discomforts, including but not limited to pain, itching, dryness, dazzling, short-term glare, and flash blindness, were reported to investigators or supervisors. The treatment was stopped immediately if the child in the intervention group had any severe adverse events, including sudden visual loss of 2 or more lines or a scotoma perceived in the center of the viewing field by the child.

Fundus images by swept-source OCT were independently evaluated by 2 accredited senior ophthalmologists. Any fundus abnormality and its grading, including but not limited to vitreomacular traction, macular schisis, macular hole, intraretinal fluid, subretinal fluid, hemorrhage, retinal pigment epithelium proliferation, and atrophy, would be recorded. Any difference between the 2 graders was adjudicated by a third senior ophthalmologist (X.X.).

### Sample Size

The sample size estimation was conducted based on the assumption of a 2-sided α level of .05, 90% power, and the expected effect size. The incidence of myopia among primary school students with premyopia was approximately 42% per year,^46,48,49^ and the expected reduction was set at 50%. This would require a total of 202 participants. Adjusting for 20% loss to follow-up yielded a total sample size of 254 participants.

### Statistical Analysis

Statistical analyses were performed using SAS, version 9.4 (SAS Institute Inc). Outcomes were analyzed by means of an intention-to-treat method and a per-protocol method. The intention-to-treat analysis included participants in both groups at baseline, while the per-protocol analysis included participants in the control group and those in the intervention group who were able to continue the intervention without interruption by the COVID-19 pandemic. A comparison between the 2 groups was performed using the χ^2^ test. The unpaired *t* test was used to compare continuous outcomes with normal distribution, while the nonparametric statistical method was used if otherwise. All *P* values were from 2-sided tests and results were deemed statistically significant at *P* < .05.

## Results

**Figure 1.**
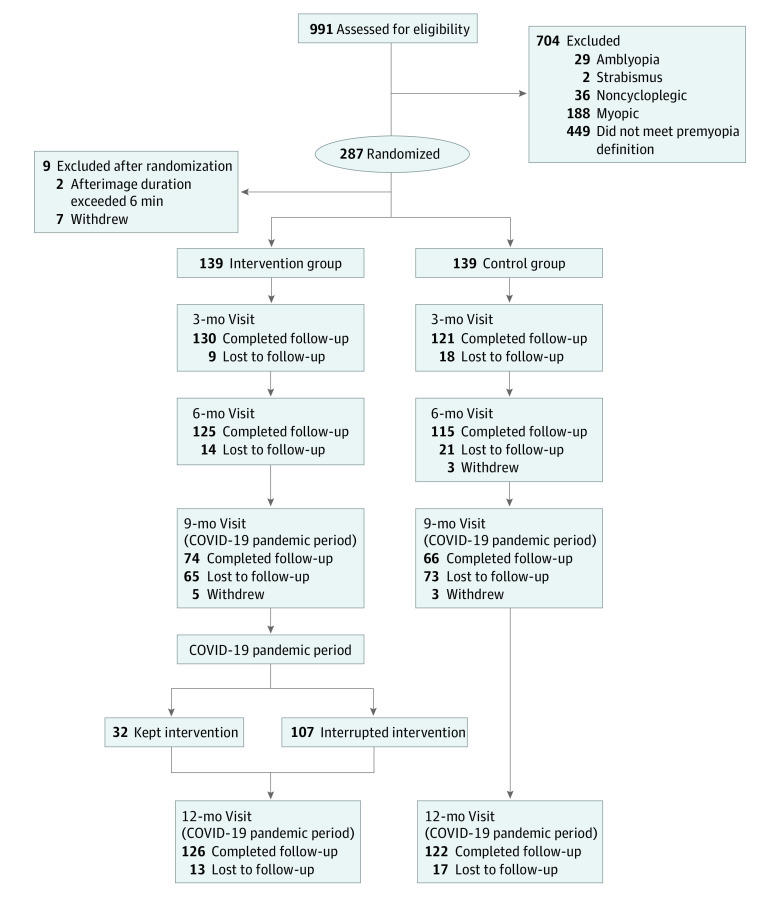
Flowchart of the Study

A total of 278 children (28.1%) were included in the trial: 139 children (mean [SD] age, 8.3 [1.1] years; 71 boys [51.1%]) in the intervention group and 139 children (mean [SD] age, 8.3 [1.1] years; 68 boys [48.9%]) in the control group (Figure 1; eTable 1 in Supplement 2). Figure 1 summarizes the number of the participants who completed enrollment, baseline examination, and intervention at each visit. Ocular characteristics, including UCVA, AL and SE, are presented in eTable 1 in Supplement 2.

Because of the COVID-19 pandemic and the associated sudden lockdown in Shanghai from March to May 2022, the number of participants at the 9-month visit and compliance with the treatment during the last 3 months in the intervention group were affected significantly when the children did not bring the devices home from school. A total of 65 of 139 children (46.8%) in the intervention group and 73 of 139 children (52.5%) in the control group did not attend the 9-month follow-up visit. For the last 3 months of the study, 32 of 139 children (23.0%) in the intervention group were able to continue the RLRL treatment. Despite this, we continued the trial and tried our best to complete the examination at the 12-month visit. Of 278 included children, 248 (89.2%) participated in the 12-month visit, consisting of 126 children (90.6%) in the intervention group and 122 children (87.8%) in the control group. The median compliance rate in the intervention group was 60.0% (IQR, 54.2%-64.8%).

### Primary Outcome

The 12-month incidence rate of myopia was 40.8% (49 of 120) in the intervention group and 61.3% (68 of 111) in the control group (Table 1). The absolute mean difference between the 2 groups was 20.4% (95% CI, 7.9%-33.1%; *P* = .003), representing a 33.4% reduction in incidence (Table 1; eFigure 2 in Supplement 2). The relative risk between the 2 groups was 0.67 (95% CI, 0.51-0.86) (Table 1). The 6-month incidence rates of myopia were 10.0% (12 of 120) for the intervention group and 22.5% (23 of 102) for the control group, indicating a 55.6% reduction in incidence (eTable 2 and eFigure 2 in Supplement 2).

**Table 1.** Refractive and Biometric Outcomes at 12-Month Follow-up (Intention-to-Treat Analysis)

Outcome	Students, % (No./total No.)		Risk difference, (95% CI)^b^	Relative risk (95% CI)^c^	Relative efficacy^d^	*P* value^e^
	Intervention^a^	Control				
Incidence of myopia, %	40.8 (49/120)	61.3 (68/111)	20.4 (7.9 to 33.1)	0.67 (0.51 to 0.86)	33.4	.003
Mean (SD), change						
SER, D	−0.35 (0.54)	−0.76 (0.60)	−0.41 (−0.56 to –0.26)	NA	53.9	<.001
AL, mm	0.30 (0.27)	0.47 (0.25)	0.17 (0.11 to 0.23)	NA	36.2	<.001

Abbreviations: AL, axial length; D, diopters; NA, not applicable; SER, spherical equivalent refraction.

^a^ The intervention group included those who continued the intervention and those with interrupted intervention.

^b^ Risk difference, absolute efficacy = value in control group − value in intervention group.

^c^ Relative risk = value in intervention group/value in control group.

^d^ Relative efficacy = (value in control group − value in intervention group)/value in control group.

^e^ The *t* test was used for change of AL and SER, and the χ^2^ test was used for incidence.

### Secondary Outcomes

For the intervention group, the mean (SD) SER change over 12 months was –0.35 (0.54) D. For the control group, the corresponding mean (SD) change was –0.76 (0.60) D. The absolute mean difference in SER change between the 2 groups was –0.41 D (95% CI, –0.56 to –0.26 D; *P* < .001) (Table 1), representing a 53.9% reduction in SER shift. The 6-month SER change for each group and the mean difference between the intervention and control groups are presented in eTable 2 in Supplement 2. Spaghetti plots of longitudinal SER data are presented in eFigure 3 in Supplement 2.

**Figure 2.**
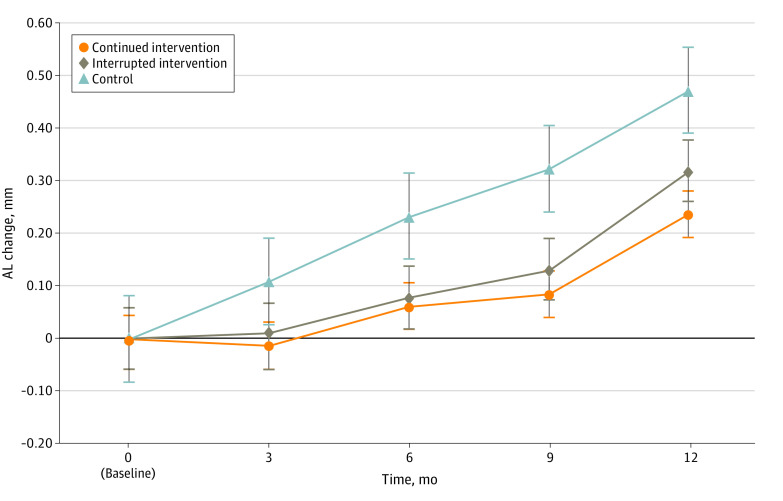
Axial Length (AL) Change Between the Intervention and Control Groups Over 12 Months

The 12-month mean (SD) AL increase was 0.30 (0.27) mm for the intervention group and 0.47 (0.25) for the control group, with an absolute mean difference in AL changes of 0.17 mm (95% CI, 0.11-0.23 mm; *P* < .001) representing a 36.2% reduction in AL changes (Table 1). The 3-, 6-, 9-, and 12- month AL changes for each group and the mean difference between the 2 groups are presented in Figure 2.

The proportions of children showing SER regression (hyperopic shift of >0.25 D, which cannot be explained as the measurement errors in refraction device) in the intervention group were 51.2% (62 of 121) at the 6-month follow-up visit and 19.0% (23 of 121) at the 12-month follow-up visit, while the corresponding proportions in the control group were 24.5% (25 of 102) at 6 months and 2.7% (3 of 111) at 12 months (eTable 3 in Supplement 2). In addition, 3.2% of children (4 of 126) in the intervention group at the 12-month follow-up achieved AL shortening of more than 0.05 mm. The proportions of children having clinically significant AL shortening in the intervention group at the 3-, 6-, and 9-month follow-up were 23.1% (30 of 130), 12.5% (15 of 120), and 9.5% (7 of 74), respectively, while the corresponding proportions in the control group were 4.1% (5 of 122), 0.9% (1 of 115), and 0%, respectively.

At the 12-month follow-up visit, the proportion of children whose UCVA decreased by at least 2 lines was significantly greater in the control group than in the intervention group (17.5% [22 of 126] vs 32.0% [39 of 122]; *P* = .02) (Table 2). The proportion of children achieving a BCVA of at least 0.8 was similar between the intervention and control groups (100.0% vs 99.2% [121 of 122]; *P* = .50). For the intervention group, the mean (SD) change in choroidal thickness over 12 months was 3.0 (16.9) μm, while for the control group, the mean (SD) change in choroidal thickness was −9.2 (22.3) μm (*P* < .001).

**Table 2.** Vision Function and Choroidal Thickness at the 12-Month Follow-up (Intention-to-Treat Analysis)

Outcome	Students, % (No./total No.)		Absolute efficacy, % (95% CI)	*P* value^b^
	Intervention^a^	Control		
Change in UCVA				
2 Lines worsening	17.5 (22/126)	32.0 (39/122)	14.5 (3.9 to 25.1)	.02
Within 1 line	77.0 (97/126)	65.6 (80/122)	11.4 (0.2 to 22.6)	
2 Lines improvement	5.6 (7/126)	2.5 (3/122)	3.1 (1.8 to 8.0)	
BCVA <0.8	0 (0/123)	0.8 (1/122)	0.8 (−0.7 to 2.3)	.50
CT change, mean (SD), μm	3.0 (16.9)	−9.2 (22.3)	12.3 (7.3 to 17.2)	<.001

Abbreviations: BCVA, best corrected visual acuity; CT, choroidal thickness; UCVA, uncorrected visual acuity.

^a^ The intervention group included those who continued the intervention and those with interrupted intervention.

^b^ The *t* test was used for CT change, and the χ^2^ test was used for change of UCVA and BCVA.

### Effect of the COVID-19 Pandemic

The baseline characteristics between the continued intervention group and the interrupted intervention group were well balanced (eTable 4 in Supplement 2). The continued intervention group had a greater reduction in the incidence rate of myopia compared with those in the control group (28.1% [9 of 32] vs 61.3% [68 of 111]) (eTable 5 in Supplement 2), representing a 54.1% reduction in incident myopia within 12 months. Similarly, the mean SER change and the mean AL change in the continued intervention group were –0.18 (0.61) D and 0.24 (0.23) mm, respectively. When compared with the mean changes in the control group, the continued intervention group achieved 76.3% and 48.9% efficacy in slowing SER and AL shifts, respectively (eTable 5 in Supplement 2).

### Adverse Events

Two participants withdrew from the study because the afterimage duration exceeded 6 minutes at baseline. According to swept-source OCT images, no other structural damage was noted in the intervention and control groups.

### Sensitivity and Subgroup Analyses

Subgroup analyses comparing the efficacy of intervention for myopia prevention and control (incidence, SER changes, and AL changes) by different baseline SER groups and age groups were performed. Better efficacy was observed among children with an SER of 0.01 to 0.50 D than among those with an SER of −0.50 to 0.00 D (relative efficacy, 64.0% vs 14.0%; SER changes, 69.0% vs 39.8%; AL changes, 45.7% vs 23.4%) (Table 3). Children of different age groups did not show significant differences in the efficacy of myopia prevention (eTable 6 in Supplement 2).

**Table 3.** Refractive and Biometric Outcomes at 12-Month Follow-up in Different Baseline SER Groups (Adjusted for Baseline Age)

Outcome and SER group	No.	Intervention^a^	Control	Absolute efficacy (relative efficacy)^b^	Difference of efficacy (95% CI)^c^
Incidence of myopia, %					
0.01 to 0.50 D	140	17.7	49.1	31.4 (64.0)	19.9 (−1.2 to 41.0)
−0.50 to 0.00 D	91	71.5	83.1	11.% (14.0)	
SER change, D					
0.01 to 0.50 D	140	−0.22	−0.71	−0.49 (69.0%)	0.17 (−0.13 to 0.46)
−0.50 to 0.00 D	91	−0.50	−0.83	−0.33 (39.8%)	
AL change, mm					
0.01 to 0.50 D	150	0.25	0.46	0.21 (45.7%)	0.10 (−0.02 to 0.23)
−0.50 to 0.00 D	98	0.36	0.47	0.11 (23.4%)	

Abbreviations: AL, axial length; D, diopters; SER, spherical equivalent refraction.

^a^ The intervention group included those who continued the intervention and those with interrupted intervention.

^b^ Absolute efficacy = value in control group − value in intervention group; relative efficacy = (value in control group − value in intervention group)/value in control group.

^c^ Difference of efficacy = the efficacy difference between 2 subgroups.

## Discussion

To our knowledge, this was the first trial to date to investigate the efficacy of the RLRL intervention in myopia prevention. In this 12-month randomized clinical trial, the RLRL intervention achieved an absolute difference of 20.4% in incidence of myopia, representing a 33.4% relative reduction in incident myopia. For children without treatment interruption due to the COVID-19 pandemic, the absolute difference increased to 33.2%, representing a 54.1% relative reduction in incident myopia over 12 months.

### Effect of RLRL Intervention on Myopia Incidence

The efficacy of a 33.4% to 54.1% relative reduction in the incidence of myopia with the RLRL intervention should be interpreted carefully. Increased outdoor time has been consistently shown to have an efficacy rate ranging from 11.0% to 54.3% in preventing myopia onset within 1 year.^11,12,13,14,50,51^ However, our study population is substantially different than those in the outdoor trials; the children without myopia at baseline were enrolled in the outdoor trials, whereas our study enrolled the children with premyopia who had a much higher risk of developing myopia. As demonstrated in our subgroup analysis, for the children with premyopia, in particular those whose SER was very close to −0.50 D (the cutoff for myopia), the prophylactic effect was much lower because the intervention was introduced too late. Considering the difference in study participants, the RLRL prophylactic effect could be even stronger if the children who had an SER of 0.01 to 0.50 D at baseline were enrolled.

### Effect of RLRL Intervention on Myopic Shift

We found that the RLRL intervention significantly reduced the myopic shifts in terms of AL and SER compared with the control group (difference, 0.17 mm and –0.41 D, respectively). In the subgroup analysis of individuals with premyopia from a randomized clinical trial in Taiwan, a 1-year outdoor intervention achieved reductions of myopia shift by 0.04 mm for AL and 0.11 D for SER.^13^ A recent study investigated the effect of 0.01% atropine on myopic shift and indicated that 0.01% atropine could reduce myopic shift by 0.09 mm in AL and 0.45 D in SER.^34^ Our study observed regression in AL (by >0.05 mm) and SER (by >0.25 D) by 3.2% and 19.0%, respectively, at 12-month follow-up, which was consistent with the report by Jiang et al^36^ of a 21.6% AL regression after an RLRL intervention among children with myopia. The effect of RLRL therapy might originate from increasing blood flow and metabolism of the fundus, as well as ameliorating scleral hypoxia, which had been proved to be a promoter for myopia development.^52,53^

### Influencing Factors of RLRL Intervention Efficacy

Our results showed that the effect of the RLRL intervention on myopia prevention was different among baseline SER groups. The RLRL intervention was more effective among children with an SER of 0.01 to 0.50 D at baseline than those with an SER of −0.50 to 0.00 D at baseline. In 2021, the International Myopia Institute defined premyopia as a refractive state of an eye of +0.75 D or less and more than −0.50 D in children.^42^ Based on our data, it might already be too late to treat those with an SER of −0.50 to 0.00 D because their incidence of myopia in 12 months could be as high as 83.1%.^42^ Therefore, prophylactic intervention, including RLRL therapy and increased outdoor time, may need to be implemented earlier, at least among those with an SER in the range of 0.01 to 0.50 D, or even earlier.

### Safety

After this 12-month RLRL intervention, no functional damage as indicated by BCVA was observed. No children reported having glare, flash blindness, or afterimages longer than 6 minutes after treatment. An afterimage induced by prior adaptation to a visual stimulus is believed to be due to bleaching of photochemical pigments or neural adaptation in the retina. Participants with an afterimage duration exceeding 6 minutes were clinically considered too sensitive to visual stimulus.^54^ No other structural changes were noted in this study, consistent with other RLRL studies.^36,40^

### Limitations

This study had several limitations. First, the open-label design may bias the results. Future studies comparing a light treatment simulator with a much lower power should exclude potential placebo effects. Second, because of the outbreak of the COVID-19 pandemic in Shanghai, approximately 70% of the children in the intervention group discontinued treatment from 9 to 12 months. Nevertheless, we tried to maximize the follow-up retention rate for the 12-month visit. Furthermore, children who continued treatment and those who discontinued treatment were well balanced for baseline characteristics. Third, the observed efficacy of the intervention in preventing myopia was generalizable only to the device used in the present study. It is unproven that other wavelengths, power intensities, or frequencies of intervention may have a similar or even better efficacy.

## Conclusions

This randomized clinical trial found that RLRL is a novel and effective intervention for myopia prevention, with good user acceptability and a 54.1% reduction in incident myopia within 12 months for children with premyopia. Our findings have public health significance, especially for myopia prevention in countries with a high incidence of myopia. More studies are needed to understand the long-term efficacy and safety, optimal intervention dose, and potential underlying mechanisms of the RLRL intervention.

## Notes

Supplement 1.
Trial Protocol

Supplement 2. **eFigure 1.** Schematic Diagram of Red Light Spectrum and Intervention Instrument **eFigure 2.** Myopia Incidence in 6- and 12-Month Between the Intervention and Control Groups **eFigure 3.** Spaghetti Plots of Longitudinal Data on SER **eTable 1.** Baseline Characteristics of the Included Participants **eTable 2.** Refractive and Biometric Outcomes at 6-Month Follow-up **eTable 3.** Percentages of SER Regression in 6 and 12 Months **eTable 4.** Baseline Characteristics Between Continued and Interrupted Intervention Groups **eTable 5.** Refractive and Biometric Outcomes at 12-Month Follow-up Between Continued Intervention and Control Group **eTable 6.** Refractive and Biometric Outcomes at 12-Month Follow-up in Different Baseline Age Groups

Supplement 3.
Data Sharing Statement
