# Errors in Abstract and Table 3

**DOI:** 10.1001/jamanetworkopen.2023.37652

**Published:** 2023-09-28

The Original Investigation titled “Effect of Repeated Low-level Red Light on Myopia Prevention Among Children in China With Premyopia: A Randomized Clinical Trial,”^1^ published on April 26, 2023, was corrected to fix an error in the total number of children with premyopia in the abstract and percentage errors in the fifth column of Table 3.
