# Supplementary material for: Effect of Repeated Low-level Red Light on Myopia Prevention Among Children in China With Premyopia: A Randomized Clinical Trial
Source: JAMA Netw Open. 2023 Apr 26;6(4):e239612. doi: 10.1001/jamanetworkopen.2023.9612 (PMC10134010; doi:10.1001/jamanetworkopen.2023.9612)
Supplement: Supplement 1. — Trial Protocol [file jamanetwopen-e239612-s001.pdf]

PROTOCOL OF PROCEDURES FOR THE REPEATED LOW-LEVEL RED-LIGHT INTERVENTION STUDY

Effect of Repeated Low-Level Red-Light on Myopia Prevention in Premyopic Children

Principal Investigators: Prof. Xun Xu and Mingguang He

Institution: Shanghai Eye Disease Prevention and Treatment Center, Shanghai, China.

Tel: +86 13386259538

Version Number: 2.0，January 2021

Table of Contents

Protocol Summary....4
1. Introduction....5
2. Methods....6
3. Interventions allocation....11
4. Data collection, management, and analysis....12
5. Monitoring....14
6. Ethics and transmission....15
7. Insurance....16
Reference....16

Protocol Summary

**Summary**	
**Purpose and Principle**	A randomized controlled trial exploring the effectiveness of low-dose single-wavelength red light on the prevention of myopia and the decrease of myopia incidence rate in children of grade 1-4 of primary schools (age of 6-11 years).
**Primary Objective**	To investigate the efficacy of low-level red-light of reducing myopia onset in premyopic children.
**Secondary Objective**	To investigate the effect of low-level red-light on spherical equivalent, axial length, uncorrected visual acuity of premyopic children.
**Study Design**	Randomized controlled trial
**Study Population**	Students of grades 1-4 in the participating schools in Shanghai
**Inclusion Criteria**	1) Students of grades 1-4 in the participating schools.<br>2) Sign informed consent to participate in screening and follow-up.<br>3) Children at a premyopic state:<br>① At least one parent with myopia (at least one eye SER <-3.0 D)<br>② -0.5D< cycloplegic SER of worse eye <=0.5D
**Exclusion Criteria**	1) Inability to obtain informed consent.<br>2) Children have astigmatism of 1.50 D or more, anisometropia of 1.50 D or more, strabismus, amblyopia, binocular vision abnormalities and other ocular abnormalities.<br>3) Children have any systemic diseases.<br>4) Children have any history of any myopia prevention interventions (e.g., low dose atropine).<br>5) Children have other reasons that the study physician considers inappropriate for inclusion in the project.
**Baseline and Follow-up of Eye Examination**	The baseline examination is in April 2021. The follow-up visits are in July 2021, October 2021, and January 2022. A total of five time points are examined in April 2022.

**Study Duration**		The baseline examination is in April 2021 , and the follow-up will be finished in April 2022. The study lasts for 12 months.
**Interventions**	**Intervention Group**	Children in the intervention group receive low-level red-light therapy 5 days per week and twice per day with each session lasting 3 minutes and an interval of at least 4 hours between.
	**Control Group**	Children in the control group do not receive low-level red-light therapy but continued their usual daily activities.
**Statistical Hypothesis**		Low-level red-light therapy can prevent the myopia onset and reduce myopia incidence among premyopic children.
**Keywords**		School-age children, low-level red-light, myopia onset prevention
**Supplementary Information**		None

1 Introduction

1.1. Study background and principle

Myopia is the most common refractive error. It has been epidemic worldwide, especially in East Asia. China has one of the highest myopia prevalence among adolescents in the world, with 15%-80%[1,2] of urban-dwelling adolescents, which is 1.5-5.6 times higher than that in Chile[3] and 11.4-30.8 times higher than that in Nepal[4]. According to the World Health Organization, myopia has become one of the leading causes of visual impairment worldwide. The high cost of optical measurement or surgery for myopia correction each year places a heavy burden on society. Preventing myopia and controlling myopia progression has become a public health problem which needs to be addressed.

In order to effectively strengthen the prevention and control of myopia among children and adolescents in China, the Ministry of Education, together with eight departments including the National Health Commission, formulated the "Implementation Plan for Comprehensive Prevention and Control of Myopia among Children and Adolescents" on 30 August 2018, and jointly with the government, schools, medical and health institutions, families and students, which aims to reduce the overall myopia prevalence among children and adolescents nationwide by more than 0.5% per year by 2023 from the 2018 level, and reduce it by more than 1% per year in provinces with a high prevalence of myopia. In addition, it is expected that by 2030, the myopia prevalence will be controlled at around 3% among 6-year-old children and will be reduced to less than 38% among primary school students, less than 60% among junior high school students, and less than 70% among senior high school students.

It is important to note that the development of myopia is a dynamic process. Myopia prevention requires intervention prior to the onset of myopia, which needs to define the "premyopia state", i.e., a refractive state that is temporarily emmetropia, with normal visual acuity and refractive error, but at a relatively high risk to develop myopia. Previous studies have shown that the risk factor scores for premyopia include the axial length growth pattern, hyperopic reserve, ocular biology, number of parental myopia, parental education, indoor/outdoor activity time and genomic risk.[5–9] The International Myopia Institute suggests that premyopia is defined as a refractive status between -0.50 D and 0.75 D in school-aged children, where age and other quantifiable risk factor scores indicate a significantly increased risk of myopia and warrant preventive intervention[10].

Interventions in children with premyopia to prevent the onset of myopia have been a long-standing medical endeavor. A retrospective study including school-aged 6-12 years old children with premyopia (defined as spherical equivalent < 1.0 D) in Taiwan, China, has demonstrated that low concentration atropine (0.025%) could effectively prevent the 1-year incidence of myopia (21% vs. 54%, P = 0.016).[11] The Singapore Phase III Atropine for the Treatment of Childhood Myopia Study (ATOM 3) has included school-aged 5-9 years old children with premyopia and low myopia (defined as spherical equivalent between -1.50 D and +1.0 D, cylinder power > -1.50 D, uncorrected visual acuity ≥ 0.8, and followed-up for 2.5 years to investigate the effectiveness of low concentration atropine (0.01%) in preventing myopia and reducing the progression of low myopia.

Low-level red-light therapy has been widely used for neurotrauma and stroke, and provides an innovative, non-invasive treatment for severe vision-impairing eye diseases such as age-related macular degeneration, amblyopia, diabetic retinopathy and Leber hereditary optic

neuropathy.[12,13] Some studies have suggested that the mechanism of low-level light therapy does not rely on the thermal effect of conventional light therapy, but rather on the photochemical conversion potential of low-level red-light, which could induce photochemical reactions in ocular tissues, increase cytochrome C oxidase activity, alter gene expression to regulate the mitochondrial respiratory chain, and increase the biological activity of nitric oxide.[14–18] We hypothesize that low-level light therapy can increase choroidal metabolic rate and circulation through the above-mentioned mechanisms, thereby improving scleral hypoxia and preventing the development and progression of myopia.

Preliminary results from the multi-center study led by our hospital suggested that low-level single-wavelength red-light was a safe intervention which did not cause detectable functional damage to the retina or macula, while significantly reducing the progression of myopia. This real-world study will for the first-time focus on typical primary schools to investigate the feasibility and effectiveness of low-level red-light therapy in reducing the prevalence of myopia in schools by preventing myopia in premyopic school-aged children. If the study is successful, it will provide new insights for comprehensive myopia prevention and control among children and adolescents.

1.2 Study purpose

Children aged 6-11 years old who meet the inclusion criteria in selected schools in Shanghai are include. A one-year randomized controlled trial is conducted to investigate the efficacy of repeated low-level red-light (RLRL) of preventing myopia onset and reducing its incidence.

1.3 Study design

To investigate the effects of RLRL on preventing myopia onset and reducing the incidence of myopia, several primary schools in Shanghai will be selected as participating schools in this study. Children from grades 1-4 in participating schools with non-cycloplegic spherical equivalent refraction (SER) < 1.0 and uncorrected visual acuity $\geq$ 0.8 are first screened based on refractive screening data. These children then answer the questionnaire and undergo cycloplegia and those with -0.5 < cycloplegic SER of worse eye <= +0.5 D and at least one parent with myopia (at least one eye SER <=-3.0 D) were included. Participants are stratified by grade and randomized into an intervention group and a control group at the ratio of 1:1.

The intervention is implemented according to the following steps: In the intervention group, subjects receive RLRL interventions in a temporary intervention room at school twice per day from Monday to Friday (each intervention lasts 3 minutes with an interval of at least 4 hours).No RLRL intervention will be performed in the control group.

For the enrolled schools, standard screenings will be completed at baseline (in April 2021). The follow-up visits are in July 2021, October 2021, and January 2022. A total of five time points are examined in April 2022. The prevalence of myopia and the effect of RLRL on preventing the onset of myopia will be determined by comparing the incidence of myopia in the intervention and control group.

2 Methods

2.1 Study site

Children aged 6-11 years who meet the inclusion criteria from grades 1-4 in selected primary schools will be enrolled as participants. Myopia examination will be conducted at 5 time points to determine the incidence and prevalence of myopia.

Temporary intervention rooms will be set up in the participating schools with a sufficient number of red-light intervention devices. Children with premyopia are identified according to the inclusion and exclusion criteria, and then randomized by grade within each school, and divided into intervention and control groups. RLRL will be implemented in the intervention group.

2.2 Inclusion criteria

1）Current students in grades 1-4 at participating schools (aged between 6 and 11 years old at baseline)

2）Sign informed consent to participate in screening and follow-up.

3）Children at a premyopic state are identified with the following diagnostic criteria:

① At least one parent with myopia (at least one eye SER <= -3.0 D)

② -0.5D < cycloplegic SER of worse ≤ 0.5D

2.3 Exclusion criteria

1) Inability to obtain informed consent.

2) Children have astigmatism of 1.50 D or more, anisometropia of 1.50 D or more, strabismus, amblyopia, binocular vision abnormalities and other ocular abnormalities.

3) Children have any systemic diseases.

4) Children have any history of any myopia prevention interventions (e.g., low dose atropine).

5) Children have other reasons that the study physician considers inappropriate for inclusion in the project.

2.4 Implementation of intervention measures

Red-light intervention:

The intervention in this study is repeated low-level single-wavelength red-light, which was based on the red-light intervention instrument (Suzhou Xuanjia Optoelectronic Technology Co., LTD., China), which is a benchtop semiconductor low-level single-wavelength red-light amblyopia interferometer. The target organ of low-level single-wavelength red-light intervention was the fundus of participants. Certified by the National Photoelectric Products Radiation Safety Quality Supervision and Inspection Center, its radiation category belongs to Class I. According to the national safety standard for laser products (GB7247.1-2012), radiation category 1 can be used safely in the eye. The product has obtained the Class II medical device registration certificate, and the manufacturer has medical device production license. The instrument is already on the market and widely used in major hospitals.

Main technical performance indicators of the intervention instrument:

1) Input power ≤100VA, input voltage: AC10V-240V, 50Hz/60Hz

2) Low-level single-wavelength red-light wavelength: 650nm±10nm

3) Diameter of low-level single-wavelength red-light cursor: 7mm±3mm, spot at the observation port: 10mm ± 2mm

4) Light source output power: 2.0mW±0.5mW; At a distance of 100mm: 1.07-1.42mw; After obtaining informed consent from the premyopic school-age children and their parents in the intervention group, the operation procedure of the intervention instrument will be explained by video, the correct method of the intervention instrument is demonstrated, and relevant instructional videos are shared for teachers, coordinators and parents to watch at any time. The premyopic school-age children in the intervention group are treated with the intervention instrument twice a day from Monday to Friday, three minutes each time, with an interval of at least 4 hours (morning break before school and afternoon break before school), under the supervision of the school teacher/coordinator in addition to routine study and life. All children have a unique corresponding personal account and password. They need to swipe the card and log in the system for verification before starting the intervention device.

Specific operation steps of low intensity single wavelength red light intervention instrument are as follows:

1) Put the instrument on a stable table, confirm the interface before connecting the accessories.

2) Connect the power supply and turn on the power switch.

3) Let the subject sit in an appropriate position, loosen the hand wheel on the side of the intervention instrument, adjust up and down to the most appropriate position, so that the eye is relatively comfortable when leaning on the eye patch, and fix the handwheel to a fixed angle.

4) The subject holds the corresponding card, swipes the card and clicks to confirm login.

5) After login, the first interface will enter the optical kinetic energy intervention item by default. Take off the glasses and click "OK". First, make sure that there is visible red light in the position of the eyes corresponding to the instrument, and then put the eyes close to the blue eye patch.

6) Before each use, please put the hands on the top of the intervention instrument and adjust the pupil distance wheel at the top. It is better to adjust two points of light to one point. If two points of light cannot be fused into one, adjust to see the brightest.

7) The optical energy will stop automatically after three minutes of use, leave the blue eye patch, close your eyes and rest for 3-5 minutes until the light spots before your eyes disappear.

8) Turn off the instrument after use.

Observation of intervention compliance:

The premyopic school-age children in the intervention group are treated in the intervention room set up in each school. Each time a card is needed to swipe into the system and start the intervention program. Therefore, the actual startup and usage can be observed in the background. In addition to the registration of the supervisor who supervises the school, another 2 project staff members are assigned to observe the intervention compliance. Monitor the usage according to the background database and conduct usage time statistics once a week. For children who use it less than 8 times per week, remind the school supervisor, and check the intervention situation of the school from time to time.

Criteria for discontinuation of observation:

The premyopic school-age children in the intervention group experienced unexpected severe adverse events (including sharp visual loss of more than two lines or central dark spots in the visual field), require withdrawal from the study, or undergo intraocular surgery midway.

2.5 Outcomes

2.5.1 Primary outcome:

1-year cumulative incidence of myopia in intervention and control groups (%)

2.5.2 Secondary outcomes

1) Spherical equivalent (SE, D) and axial length progression in the intervention group and control group
2) Changes of uncorrected visual acuity (UCVA) and best corrected visual acuity (BCVA) in the intervention group and the control group
3) Changes of choroid thickness (CT) in the intervention group and the control group

2.5.3 Definition of myopia:

A cycloplegic SER (sphere plus half of cylinder) $\leq$ -0.50 D.

2.5.4 Side effects:

Adverse events, e.g., afterimage duration exceeded 6 minutes, eye discomforts including but not limited to pain, itching, dryness, dazzling, short-term glare and flash blindness, severe adverse events including sudden visual loss of $\geq$2 lines or a scotoma perceived by the children in the center of the viewing field.

2.6 Subject schedule

The study will complete the baseline examination in April 2021. The follow-up visits are in July 2021, October 2021, and January 2022. A total of five time points are examined in April 2022.

2.7 Work procedures

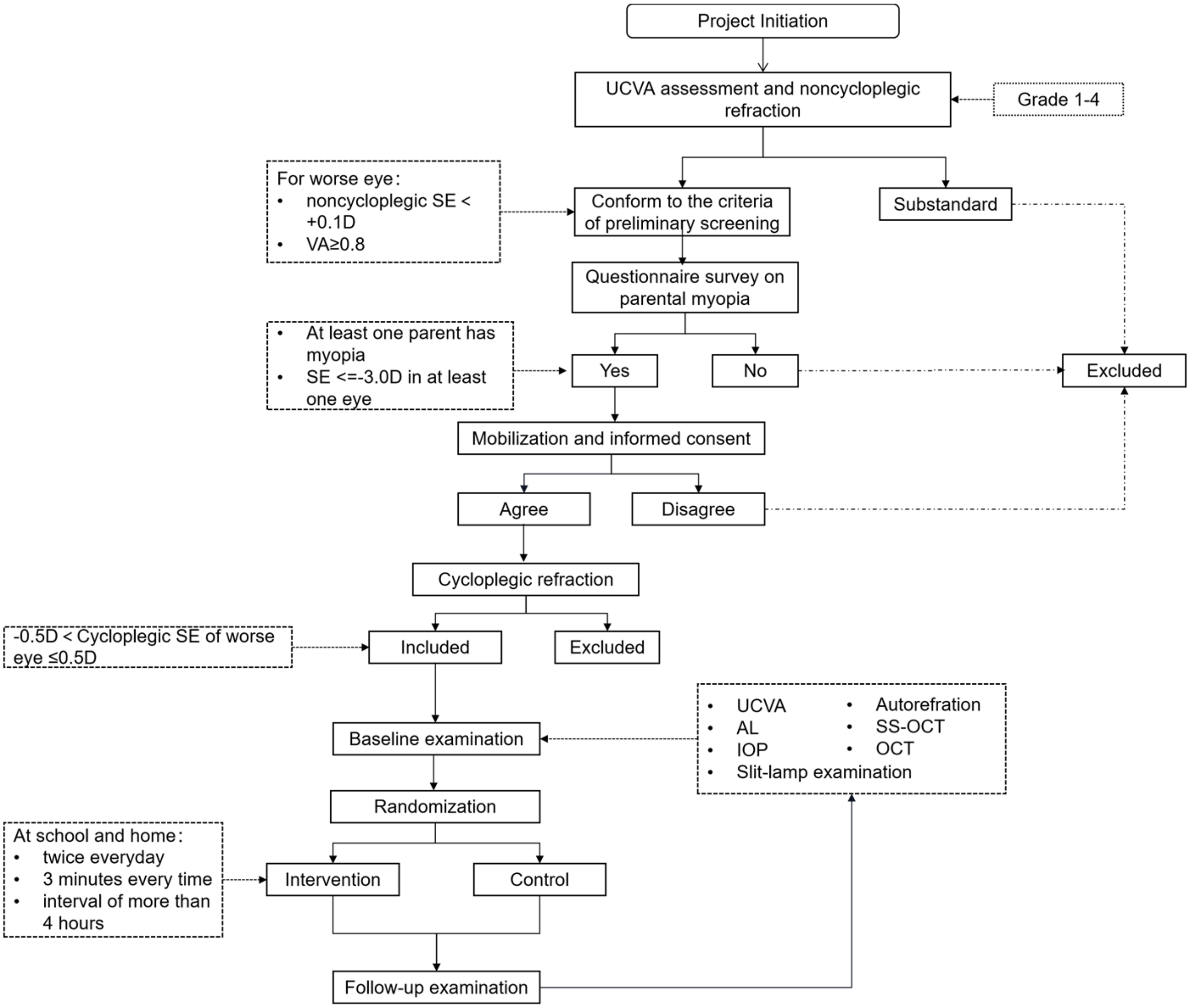
Project Initiation
UCVA assessment and noncycloplegic refraction
Grade 1-4
For worse eye:
• noncycloplegic SE < +0.1D
• VA≥0.8
Conform to the criteria of preliminary screening
Substandard
Questionnaire survey on parental myopia
• At least one parent has myopia
• SE <=-3.0D in at least one eye
Yes
No
Excluded
Mobilization and informed consent
Agree
Disagree
Cycloplegic refraction
-0.5D < Cycloplegic SE of worse eye ≤0.5D
Included
Excluded
• UCVA
• AL
• IOP
• Slit-lamp examination
• Autorefration
• SS-OCT
• OCT
Baseline examination
Randomization
At school and home:
• twice everyday
• 3 minutes every time
• interval of more than 4 hours
Intervention
Control
Follow-up examination

2.8 Compliance management

The coordinator shall uniformly manage and solve the problems encountered by students during operation, and at the same time supervise and record the relevant interventions. Through the user account, count the number of backstage usage every week, carry out joint supervision with the coordinator, and randomly check the work of the coordinator to urge him to complete the recording and coordination work on time and quality.

2.9 Sample size calculation

This study is a real-world study based on selected elementary schools in Shanghai, and the sample size was calculated according to the district group randomization formula with the following parameter settings: according to the data from previous studies, the average annual myopia incidence for elementary school students in grades 1-4 is 42%, and it is expected that the use of low-level single-wavelength red-light in schools can reduce the incidence of myopia by 50%, i.e., the incidence of myopia decreases to 21%. A total of 202 students are required based on a test efficacy of 90% and $\alpha=0.05$. Considering a participation rate of 90% and a missed visit rate of less than 10% per year, a total of 254 individuals would be required.

2.10 Recruitment

Some primary school children in Shanghai will be invited to participate in this study. First, school-age children in grades 1-4 in participating schools will be screened for non-cycloplegic spherical equivalent (SE) less than 1.0 D and UCVA $\geq$ 0.8 based on refractive developmental profile data and undergo cycloplegia. Those with -0.5 D < cycloplegic SE of worse eye ≤ +0.5 D and at least one parent has myopia (SER <=-3.0D) will be selected and the intervention and control groups were formed according to the intra-school matched-grade individual randomization method.

3 Interventions allocation

3.1 Randomization

To avoid bias, The study will use a computer system centrally controlled randomization method to achieve central randomized grouping.

3.2 Allocation concealment

After the investigators in each center access the server through the web and enter the information of individuals, the computerized randomization system will divide the individuals according to the random assignment table and lock the division information of individuals in the system.

3.3 Allocation implementation

In the central randomization system, the statistical expert in charge of randomization designs the randomization parameters in advance in the background, and the system generates the random assignment table.

3.4 Blinding

In this study, the grouping and other information will be concealed from the eye examiners and data analysts, and a single blind is set up.

4 Data collection, management, and analysis

4.1 Data collection

The examination consists of one baseline and four follow-up examinations. Detailed content or description are described as follows:

1) Registration: Name, date of birth, gender, ethnicity, contact information, school and class information.

2) Questionnaire (for follow-ups only): Subjective questions on discomfort after intervention.

3) Distant visual acuity test: ETDRS Log MAR E visual acuity chart (Precision Vision, Villa Park, Illinois, USA) is used to test binocular uncorrected visual acuity by trained personnel.

4) Axial length measurement: IOL Master (version 5.02, Carl Zeiss Meditec, Germany) is used for axial length measurement. Calibrate with a simulated eye before measurement. Each eye is measured 3 times to have the average value obtained, and the difference between each two values should be less than 0.02 mm. Those with large fluctuations despite multiple measurements is recorded.

5) Intraocular pressure (IOP) measurement: non-contact tonometer (NT-4000, Nidek Company, USA) is used to test IOP. Each eye is measured 3 times with the average value obtained. The difference between each two values should be less than 5 mmHg. Those higher than 24 mmHg should be recorded, and visual field examination should be done. Details of visual field inspection are to be determined.

6) Ophthalmologist examination: Ophthalmologist examination consists of anterior segment slit lamp examination (66 Vision. Tech, Suzhou) and ophthalmoscopy (66 Vision. Tech, Suzhou). The anterior segment slit lamp examination includes examination of eyelids, conjunctiva, cornea, anterior chamber, iris, pupil, lens, and anterior vitreous body, and is performed by senior experienced ophthalmologists. Those with peripheral anterior chamber depth less than 1/2 corneal thickness, or those who have acute anterior segment inflammation, or other related diseases, and are not suitable for further cycloplegia, will be recorded. Either will the examination be suspended, or they be excluded from this study. In addition, methods such as corneal light reflection, cover-uncover, etc. are used for strabismus detection and typing (phoria or manifest strabismus). Those with manifest strabismus will be excluded. The eyelids, conjunctiva, cornea, sclera, iris, lens, vitreous body and fundus retina are checked for abnormalities and changes.

7) Cycloplegia: Instill 1 drop of 0.5% proparacaine (Alcaine, Alcon Company) into the conjunctival sac of each eye, followed by 2 drops of 1% cyclopentolate (Cyclogyl, Alcon), with 5 minutes between each drop. After each drip, instruct the student to press the inner canthus softly for a few seconds, and try to adopt the posture of leaning his head back. Check light reflex 30 minutes after the last drop of cyclopentolate. Cycloplegia is considered complete if the light reflex disappears and the pupil diameter exceeds 6 mm. A third drop of cyclopentolate is needed if the light reflex still exists. Recheck the light reflex and pupil diameter after 20 minutes. Record if light reflex persists. Any eye discomfort should be carefully examined and corresponding treatment should be given by the ophthalmologist during the process of

cycloplegia.
8) Automatic optometry test: Automatic optometry (Topcon KR8800) is used to measure the spherical diopter, cylindrical diopter, and axial direction of both eyes. A complete inspection is done according to the standard operating requirements of the autorefractor.
9) Swept-source OCT (SS-OCT) examination: SS-OCT (DRI OCT Triton, Topcon, Tokyo, Japan) examination is performed after cycloplegia. Imaging site: macula + optic disc area, mode: 12*9 mm 3D scan mode (4 overlap)/Line scan (64 overlap) + macular area 9 mm radial scan mode (16 overlap, follow up mode) + optic disc area 9 mm radial scan mode (16 overlap, follow up mode) + macular area 7*7 mm 3D scan mode (4 overlap) + optic disc area 6*6 mm 3D scan mode (4 overlap). Requirements: Input spherical lens, cylindrical lens, eye axis and corneal curvature radius correction magnification before imaging; image signal strength of 3D scan mode should not be lower than 50, radial scan mode 60; avoid flip mirrors on the image of the peripheral area; manually adjust the capture center to the center of the optic disc when imaging the optic disc area; re-image if the quality is affected by blinking or eye movement during the process. Record if satisfying images fail to be obtained after multiple tries. The SS-OCT comes with an innate function of color fundus photography. When photographing, the imaging site is required to be consistent with the SS-OCT scanning site. Avoid eyelids, eyelashes or hair covering, and dark areas in the image, as well as a lower-than-90 image quality.

4.2 Data management

A paper form was used during the examination to complete the registration of the subject's personal information, record the completeness of the examination process, and the device printed the results and attached them to the examination form as required. Data collected on site is entered instantly using the clinical data platform (EDC) system, ensuring that all required fields are entered and submitted after the system indicates that there are no missing values. The raw data captured by the equipment will be uploaded simultaneously via the collection device provided by the project. Upon completion of the examination, paper examination forms will be kept in a locked cabinet in the research center to protect the privacy of the participants and to locate and check the raw data.

4.3 Statistical analysis plan

The aim of this study was to investigate the effect of low-level single-wavelength red-light on prevent myopia and reduce the incidence of myopia in schools. Myopia in this study is defined as equivalent spherical (SE) $\leq$ -0.50 D based on cycloplegic autorefraction results.

Data analysis will be performed under the intention-to-treat (ITT) and and per-protocol (PP) principle. All subjects who complete the protocol and those who fail to complete the protocol but do not withdraw from the study will be included in the analysis. For subjects who withdraw from the study, valid data collected before withdrawal will be included in the analysis data set.

The primary outcome is the incidence of myopia, and secondary outcomes are the progression of SE, AL, UCVA, BCVA, and CT.

Descriptive statistics: Continuous variables: sample size, mean, standard deviation, minimum, maximum, quartile.
Categorical variables: frequency distribution.

To compare the incidence of myopia in the intervention and control groups, a chi-square test will be used. For statistical analysis based on other secondary outcomes, the data from the worse eye will be taken for analysis. If quantitative traits conform to a normal distribution, the significance will be tested according to an unpaired t-test. If they do not conform to a normal distribution, the significance will be tested by non-parametric statistical methods or by taking cut-off points according to clinical significance, calculating the proportions of categorical variables and testing significance by chi-square tests.

Sensitivity analyses based on the protocol strategy will be performed to investigate the efficacy of repeated low-level red light therapy on primary outcome and secondary outcomes. Sensitivity analyses will be further performed to assess efficacy of repeated low-level red-light therapy across different refraction groups and age groups.

5 Monitoring

5.1 Data Monitoring

An independent Data and Safety Monitoring Committee was established. all of the members are independent of the project sponsor and have no conflict of interest. Members of the Data and Safety Committee regularly review the data collection process, storage, and analysis, and have access to the raw data associated with this clinical study to determine the completeness, accuracy and consistency of the information with the original data holdings. Relevant information needs to be readily available to members of the Data and Safety Committee. The Data and Safety Committee Board is required to review all data and informed consent.

5.2 Risks/hazards (adverse reactions)

The intervention used in this study is the Eyersing Amblyopia Intervention Device, which has received a national safety test certificate, and a Class II medical device registration certificate, and the manufacturer has a medical device manufacturing license. The intervention device has been marketed and is widely used in major hospitals. It has been used clinically for more than ten years in the field of amblyopia treatment and has no known side effects on the structural function of the eye. The intervention device uses a diode to produce a single wavelength of visible red light at 650±10 nm, reaching the eye with an energy range of 1.07 to 1.42 mw. It has been certified by the National Centre for Radiation Safety and Quality Supervision and Inspection of Optoelectronic Products as Class I radiation. According to the document GB 7247.1-2012 "Safety of Radiation Products Part I: Equipment Classification, Requirements", radiation is safe for use in the eye. After using the intervention device, there may be a temporary reaction of dazzling, flash blindness, and post-optical light spots, which may resolve themselves after a few minutes of rest with eyes closed.

5.3 Audit

An auditing team independent of the sponsor is set up by the investigator to visit each school

study site 1-2 weeks at the start of the project to provide guidance and training and to review the progress of the trial implementation.

6 Ethics and transmission

6.1 Ethical approval

Apply to ethics committees of Shanghai General hospital for review and approval. The protocol is implemented after approval of ethics committee and the procedure should meet the regulations and requirements of responsible institutions. Once it`s approved, the adjusted protocol should be reapproved by ethics committee before its implementation.

6.2 Protocol adjustment

Adjustments of protocol should be reviewed and approved by sponsors and ethics committee before its implementation, unless it is intended to eliminate the emergent harm to the subject, or it's only about administrative management and/or logic concerns of the study (such as changing phone number).

6.3 Informed consent

Procedures and documentation in the study should be reviewed and approved by ethics committee before use. The process of informed consent provides continuous explanations so that they can fully understand and consider before deciding whether to start or continue the study. Researchers will discuss the content of the study with the subjects and their parents/guardians. Subjects and their parents/guardians are able to ask questions before, during and after study. Subjects and their parents/guardians have the right to be informed during the study and can quit the study without reasons anytime.

The informed consent of participants provides the scheme of the study, including purpose of the study, procedures and scheduled plans, potential risks, and benefits as well as alternative interventions. The informed consent also explains the rights once the subjects participate in the study. The subjects should be given enough time to consider whether to participate in the study, and if they agree to be volunteers, they need to sign the informed consent.

6.4 Secrecy

According to agreements, all the involved must keep confidential during the trial. All data related cannot be visited without permission. Private information of participants will be protected in reports and all publications of clinical research data.

6.5 Interest statement

The people in charge of the trial have no financial or other conflicts of interest.

6.6 Data collection

Statements about who have access to the final database and disclosure of the contract restricting researchers from obtaining final data of the trial.

6.7 Subsidiary and care after trial

This study has no subsidiary and care after trial.

6.8 Communication policy

In the last follow up, we will offer every participant a report describing their random state, final refractive state, and comparison of refractive state before and after the trial. Any subsequent publications will be provided to every participant who request it for free. Results of the study will be published in public as part of the papers of research team and can be obtained in the library.

7 Insurance

Accident insurance of clinical trial will be provided to all children enrolled by researchers.

Reference

1. He M, Zeng J, Liu Y, Xu J, Pokharel GP, Ellwein LB. Refractive error and visual impairment in urban children in southern china. *Invest Ophthalmol Vis Sci*. 2004;45(3):793-799. doi:10.1167/iovs.03-1051
2. Zhao J, Pan X, Sui R, Munoz SR, Sperduto RD, Ellwein LB. Refractive Error Study in Children: results from Shunyi District, China. *Am J Ophthalmol*. 2000;129(4):427-435. doi:10.1016/s0002-9394(99)00452-3
3. Maul E, Barroso S, Munoz SR, Sperduto RD, Ellwein LB. Refractive Error Study in Children: results from La Florida, Chile. *Am J Ophthalmol*. 2000;129(4):445-454. doi:10.1016/s0002-9394(99)00454-7
4. Pokharel GP, Negrel AD, Munoz SR, Ellwein LB. Refractive Error Study in Children: results from Mechi Zone, Nepal. *Am J Ophthalmol*. 2000;129(4):436-444. doi:10.1016/s0002-9394(99)00453-5
5. Mutti DO, Hayes JR, Mitchell GL, et al. Refractive error, axial length, and relative peripheral refractive error before and after the onset of myopia. *Invest Ophthalmol Vis Sci*. 2007;48(6):2510-2519. doi:10.1167/iovs.06-0562
6. Zadnik K, Sinnott LT, Cotter SA, et al. Prediction of Juvenile-Onset Myopia. *JAMA Ophthalmol*. 2015;133(6):683-689. doi:10.1001/jamaophthalmol.2015.0471
7. Zhang M, Gazzard G, Fu Z, et al. Validating the accuracy of a model to predict the onset of myopia in children. *Invest Ophthalmol Vis Sci*. 2011;52(8):5836-5841. doi:10.1167/iovs.10-5592
8. Jones-Jordan LA, Sinnott LT, Manny RE, et al. Early childhood refractive error and

parental history of myopia as predictors of myopia. *Invest Ophthalmol Vis Sci*. 2010;51(1):115-121. doi:10.1167/iovs.08-3210

9. Tedja MS, Wojciechowski R, Hysi PG, et al. Genome-wide association meta-analysis highlights light-induced signaling as a driver for refractive error. *Nat Genet*. 2018;50(6):834-848. doi:10.1038/s41588-018-0127-7

10. Flitcroft DI, He M, Jonas JB, et al. IMI - Defining and Classifying Myopia: A Proposed Set of Standards for Clinical and Epidemiologic Studies. *Invest Ophthalmol Vis Sci*. 2019;60(3):M20-M30. doi:10.1167/iovs.18-25957

11. Fang PC, Chung MY, Yu HJ, Wu PC. Prevention of myopia onset with 0.025% atropine in premyopic children. *J Ocul Pharmacol Ther*. 2010;26(4):341-345. doi:10.1089/jop.2009.0135

12. Bjordal JM, Couppé C, Chow RT, Tunér J, Ljunggren EA. A systematic review of low level laser therapy with location-specific doses for pain from chronic joint disorders. *Aust J Physiother*. 2003;49(2):107-116. doi:10.1016/s0004-9514(14)60127-6

13. Peplow PV, Chung TY, Baxter GD. Laser photobiomodulation of wound healing: a review of experimental studies in mouse and rat animal models. *Photomed Laser Surg*. 2010;28(3):291-325. doi:10.1089/pho.2008.2446

14. Karu TI, Pyatibrat LV, Afanasyeva NI. A novel mitochondrial signaling pathway activated by visible-to-near infrared radiation. *Photochem Photobiol*. 2004;80(2):366-372. doi:10.1562/2004-03-25-RA-123

15. Karu TI, Pyatibrat LV, Kolyakov SF, Afanasyeva NI. Absorption measurements of a cell monolayer relevant to phototherapy: reduction of cytochrome c oxidase under near IR radiation. *J Photochem Photobiol B*. 2005;81(2):98-106. doi:10.1016/j.jphotobiol.2005.07.002

16. Lohr NL, Keszler A, Pratt P, Bienengraber M, Warltier DC, Hogg N. Enhancement of nitric oxide release from nitrosyl hemoglobin and nitrosyl myoglobin by red/near infrared radiation: potential role in cardioprotection. *J Mol Cell Cardiol*. 2009;47(2):256-263. doi:10.1016/j.yjmcc.2009.03.009

17. Posten W, Wrone DA, Dover JS, Arndt KA, Silapunt S, Alam M. Low-level laser therapy for wound healing: mechanism and efficacy. *Dermatol Surg*. 2005;31(3):334-340. doi:10.1111/j.1524-4725.2005.31086

18. Zhang Y, Song S, Fong CC, Tsang CH, Yang Z, Yang M. cDNA microarray analysis of gene expression profiles in human fibroblast cells irradiated with red light. *J Invest Dermatol*. 2003;120(5):849-857. doi:10.1046/j.1523-1747.2003.12133.x
