# Supplementary material for: Effect of Repeated Low-level Red Light on Myopia Prevention Among Children in China With Premyopia: A Randomized Clinical Trial
Source: JAMA Netw Open. 2023 Apr 26;6(4):e239612. doi: 10.1001/jamanetworkopen.2023.9612 (PMC10134010; doi:10.1001/jamanetworkopen.2023.9612)
Supplement: Supplement 2. — eFigure 1. Schematic Diagram of Red Light Spectrum and Intervention Instrument eFigure 2. Myopia Incidence in 6- and 12-Month Between the Intervention and Control Groups eFigure 3. Spaghetti Plots of Longitudinal Data on SER eTable 1. Baseline Characteristics of the Included Participants eTable 2. Refractive and Biometric Outcomes at 6-Month Follow-up eTable 3. Percentages of SER Regression in 6 and 12 Months eTable 4. Baseline Characteristics Between Continued and Interrupted Intervention Groups eTable 5. Refractive and Biometric Outcomes at 12-Month Follow-up Between Continued Intervention and Control Group eTable 6. Refractive and Biometric Outcomes at 12-Month Follow-up in Different Baseline Age Groups [file jamanetwopen-e239612-s002.pdf]

Supplementary Online Content

He X, Wang J, Zhu Z, et al. Effect of repeated low-level red light on myopia prevention among children in China with premyopia: a randomized clinical trial. *JAMA Netw Open.* 2023;6(4):e239612. doi:10.1001/jamanetworkopen.2023.9612

eFigure 1. Schematic Diagram of Red Light Spectrum and Intervention Instrument
eFigure 2. Myopia Incidence in 6- and 12-Month Between the Intervention and Control Groups
eFigure 3. Spaghetti Plots of Longitudinal Data on SER
eTable 1. Baseline Characteristics of the Included Participants
eTable 2. Refractive and Biometric Outcomes at 6-Month Follow-up
eTable 3. Percentages of SER Regression in 6 and 12 Months
eTable 4. Baseline Characteristics Between Continued and Interrupted Intervention Groups
eTable 5. Refractive and Biometric Outcomes at 12-Month Follow-up Between Continued Intervention and Control Group
eTable 6. Refractive and Biometric Outcomes at 12-Month Follow-up in Different Baseline Age Groups

This supplementary material has been provided by the authors to give readers additional information about their work.

© 2023 He X et al. *JAMA Network Open.*

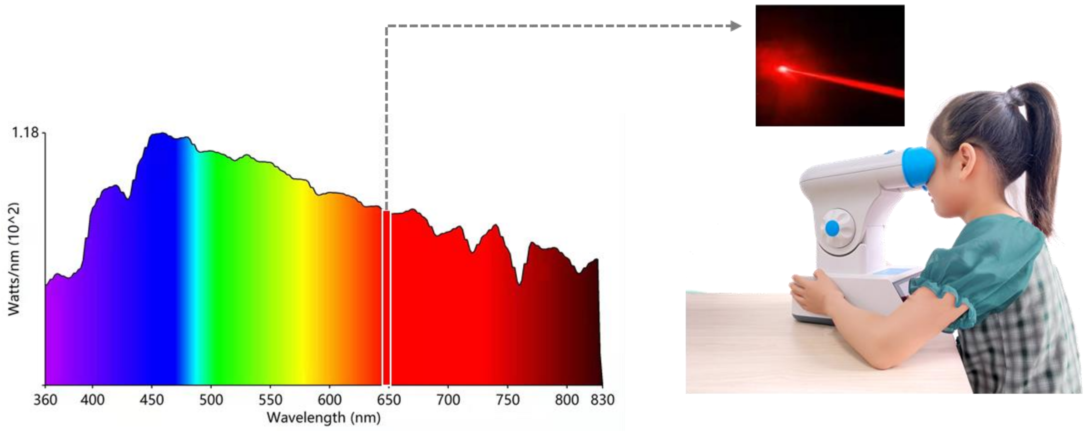

eFigure 1. Schematic Diagram of Red Light Spectrum and Intervention Instrument

© 2023 He X et al. *JAMA Network Open.*

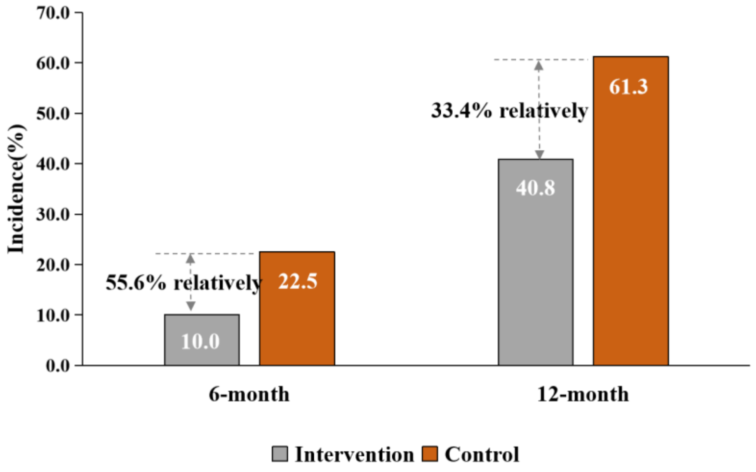

eFigure 2. Myopia Incidence in 6- and 12-Month Between the Intervention and Control Groups

© 2023 He X et al. *JAMA Network Open.*

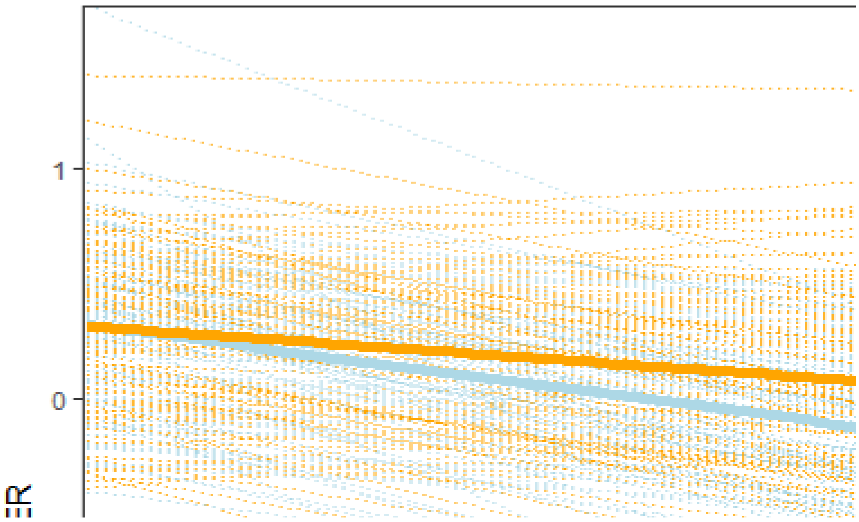

eFigure 3. Spaghetti Plots of Longitudinal Data on SER

© 2023 He X et al. *JAMA Network Open.*

eTable 1. Baseline Characteristics of the Included Participants

Characteristics	Intervention (N=139)	Control (N=139)
Age, mean (SD), yrs	8.28 (1.10)	8.31 (1.07)
Gender (boys)		
boys	51.1% (71/139)	48.9% (68/139)
girls	48.9% (68/139)	51.1% (71/139)
UCVA		
Mean (SD)	0.8 (0.2)	0.8 (0.2)
Median (P25-P75)	0.8 (0.8,1.0)	0.8 (0.8,1.0)
AL, mm		
Mean (SD)	23.36 (0.68)	23.30 (0.69)
Median (P25-P75)	23.37 (22.97,23.82)	23.25 (22.80,23.71)
SER, D		
Mean (SD)	0.14 (0.30)	0.16 (0.28)
Median (P25-P75)	0.25 (-0.13,0.38)	0.25 (-0.13,0.38)

Abbreviations: UCVA, uncorrected visual acuity; AL, axial length; SER, spherical equivalent refraction; SD, standard deviation.

© 2023 He X et al. *JAMA Network Open.*

eTable 2. Refractive and Biometric Outcomes at 6-Month Follow-up

	Intervention	Control	Absolute efficacy (Relative efficacy)	P value
Incidence	10.0% (12/120)	22.5% (23/102)	12.5% (55.6%)	0.018*
Change, mean (SD)				
SER (D)	0.18 (0.35)	-0.14 (0.46)	-0.32 (228.6%)	<0.001*
AL (mm)	0.07 (0.13)	0.23 (0.16)	0.16 (69.6%)	<0.001*

SER, spherical equivalent refraction; AL, axial length; SD, standard deviation

Absolute efficacy = value in control group - value in intervention group

Relative efficacy = (value in control group - value in intervention group)/value in control group

* t-test for change of AL and SE; chi-square test for incidence.

© 2023 He X et al. *JAMA Network Open.*

eTable 3. Percentages of SER Regression in 6 and 12 Months

	Intervention	Control	P value
6M			
>=0.25D	51.2% (62/121)	24.5% (25/102)	<0.001*
>=0.50D	22.3% (27/121)	6.9% (7/102)	0.003*
>=0.75D	6.6% (8/121)	2.9% (3/102)	0.342
12M			
>=0.25D	19.0% (23/121)	2.7% (3/111)	0.001*
>=0.50D	5.0% (6/121)	1.8% (2/111)	0.284
>=0.75D	0.8% (1/121)	0.0% (0/111)	1.000

* chi-square test for proportion between the two groups

© 2023 He X et al. *JAMA Network Open.*

eTable 4. Baseline Characteristics Between Continued and Interrupted Intervention Groups

Characteristics	Continued intervention (N=32)	Interrupted intervention (N=107)	Control (N=139)
Age, mean (SD)	8.27 (1.10)	8.29 (1.11)	8.31 (1.07)
Gender			
Boys	14 (43.8%)	57 (53.3%)	48.9% (68/139)
Girls	18 (56.2%)	50 (46.7%)	51.1% (71/139)
UCVA			
Mean (SD)	0.8 (0.1)	0.8 (0.2)	0.8 (0.2)
Medium (P25-P75)	0.8(0.8,1.0)	0.8(0.8,1.0)	0.8 (0.8,1.0)
AL			
Mean (SD)	23.28 (0.62)	23.39 (0.70)	23.30 (0.69)
Medium (P25-P75)	23.27(23.02, 23.61)	23.40(22.95,23.87)	23.25 (22.80,23.71)
SER			
Mean (SD)	0.13 (0.31)	0.14 (0.30)	0.16 (0.28)
Medium (P25-P75)	0.25(-0.16,0.38)	0.25(-0.13,0.38)	0.25 (-0.13,0.38)

UCVA, uncorrected visual acuity; AL, axial length; SER, spherical equivalent refraction; SD, standard deviation.

Comparison between either two of the three groups showed no significance.

© 2023 He X et al. *JAMA Network Open.*

eTable 5. Refractive and Biometric Outcomes at 12-Month Follow-up Between Continued Intervention and Control Group

Variables	Continued intervention (N=32)	Control (N=139)	Risk difference (95% CI)	Relative risk (95% CI)	Relative efficacy	P value [a]
Incidence of myopia, %	28.1% (9/32)	61.3% (68/111)	33.2% (15.2%, 51.2%)	0.46 (0.26, 0.81)	54.1%	0.002
Change, mean (SD)						
SER, D	-0.18 (0.61)	-0.76 (0.60)	-0.58 (-0.82, -0.34)	-	76.3%	<0.001
AL, mm	0.24 (0.23)	0.47 (0.25)	0.23 (0.14, 0.32)	-	48.9%	<0.001

Abbreviations: SER, spherical equivalent refraction; AL, axial length, SD, standard deviation; CI, confidence interval

Risk difference = Absolute efficacy = value in control group - value in intervention group

Relative risk = value in intervention group / value in control group

Relative efficacy = (value in control group - value in intervention group)/value in control group

[a] t-test for change of AL and SE; chi-square test for incidence.

© 2023 He X et al. *JAMA Network Open.*

eTable 6. Refractive and Biometric Outcomes at 12-Month Follow-up in Different Baseline Age Groups

Variables	N	Intervention	Control	Absolute efficacy (Relative efficacy)	Difference of efficacy (95%CI)
Incidence of myopia, %					
baseline 6-8 yrs	109	49.2% (29/59)	66.0% (33/50)	16.9% (25.5%)	
baseline 8-11 yrs	122	32.8% (20/61)	57.4% (35/61)	24.6% (42.9%)	-7.7% (-32.8%, 17.3%)
SER change (D)					
baseline 6-8 yrs	109	-0.46 (0.53)	-0.89 (0.68)	-0.43 (48.3%)	0.02 (-0.28, 0.31)
baseline 8-11 yrs	122	-0.24 (0.53)	-0.65 (0.52)	-0.41 (63.1%)	
AL change (mm)					
baseline 6-8 yrs	115	0.35 (0.22)	0.59 (0.27)	0.24 (40.7%)	0.10 (-0.03, 0.23)
baseline 8-11 yrs	133	0.25 (0.32)	0.39 (0.19)	0.14 (35.9%)	

SER, spherical equivalent refraction; AL, axial length, SD, standard deviation

Absolute efficacy = value in control group - value in intervention group

Relative efficacy = (value in control group - value in intervention group)/value in control group

© 2023 He X et al. *JAMA Network Open.*
