# Supplementary material for: Effect of Repeated Low-level Red Light on Myopia Prevention Among Children in China With Premyopia: A Randomized Clinical Trial
Source: JAMA Netw Open. 2023 Apr 26;6(4):e239612. doi: 10.1001/jamanetworkopen.2023.9612 (PMC10134010; doi:10.1001/jamanetworkopen.2023.9612)
Supplement: Supplement 3. — Data Sharing Statement [file jamanetwopen-e239612-s003.pdf]

Data Sharing Statement

He. Effect of Repeated Low-Level Red Light on Myopia Prevention Among Children in China With Premyopia. *JAMA Netw Open*. Published April 26, 2023.
doi:10.1001/jamanetworkopen.2023.9612

Data
Data available: No

Additional Information
Explanation for why data not available: Data will be shared upon reasonable request to pursue additional studies or for replication.
